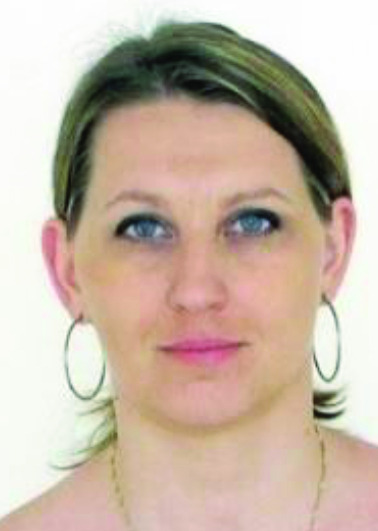# Fluorescence‐Based HTS Assays for Ion Channel Modulation in Drug Discovery Pipelines

**DOI:** 10.1002/cmdc.202400383

**Published:** 2024-10-29

**Authors:** Jan Voldřich, Marika Matoušová, Markéta Šmídková, Helena Mertlíková‐Kaiserová

**Affiliations:** ^1^ Institute of Organic Chemistry and Biochemistry Academy of Sciences of the Czech Republic Flemingovo nam. 2 Prague 6 – Dejvice 16610 Czech Republic; ^2^ University of Chemistry and Technology Technická 5 Prague 6 – Dejvice 166 28 Czech Republic

**Keywords:** Fluorescence-based assay, Fluorescent probes, High-throughput screening, Ion channels

## Abstract

Ion channels represent a druggable family of transmembrane pore‐forming proteins with important (patho)physiological functions. While electrophysiological measurement (manual patch clamp) remains the only direct method for detection of ion currents, it is a labor‐intensive technique. Although automated patch clamp instruments have become available to date, their high costs limit their use to large pharma companies or commercial screening facilities. Therefore, fluorescence‐based assays are particularly important for initial screening of compound libraries. Despite their numerous disadvantages, they are highly amenable to high‐throughput screening and in many cases, no sophisticated instrumentation or materials are required. These features predispose them for implementation in early phases of drug discovery pipelines (hit identification), even in an academic environment. This review summarizes the advantages and pitfalls of individual methodological approaches for identification of ion channel modulators employing fluorescent probes (i. e., membrane potential and ion flux assays) with emphasis on practical aspects of their adaptation to high‐throughput format.

## Graphical abstract

This review focuses on the latest methods for screening ion channel modulators using fluorescent probes. The methods covered include cell‐based membrane potential assays and ion flux assays. Their benefits and drawbacks are highlighted, with an emphasis on practical aspects and use in high‐throughput screening. Moreover, suitable cell models utilized in these methods and their preparation are discussed.

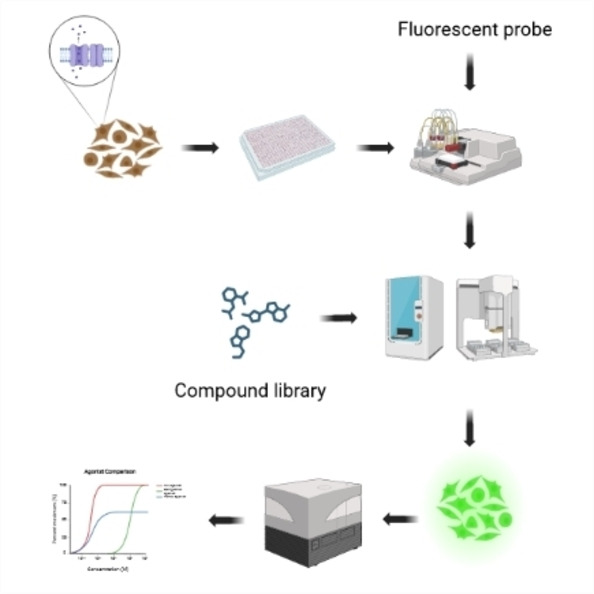

## 1 Introduction

Ion channels are membrane proteins that regulate many physiological processes, including cell signaling, proliferation, secretion or membrane potential. This makes them frequent drug targets −18 % of the small molecule drugs found in ChEMBL database have been described to target ion channels.[^1^, ^2^] On the other hand, ion channels may easily become off‐targets, with the hERG channel probably being the most prominent example of a potassium channel whose blockage leads to undesired toxicity.^[3]^

The primary function of ion channels is to mediate permeation of ions across biological membranes. Due to the hydrophobic character of an inner part of lipid bilayer, the membranes function as natural barriers for very polar and/or charged molecules such as ions. Ion channels form narrow hydrophilic pores that can close and open and allow specific inorganic ions (primarily for Na^+^, K^+^, Ca^2+^, or Cl^−^) to move down the electrochemical gradient.^[4]^

The methods that are currently available for screening of ion channel modulators could be divided into functional and non‐functional. Non‐functional methods detect binding affinity of tested compound to a channel rather than the effect of the compound on the channel's activity. An example of this type of assay is a radioligand binding assay, which can be easily optimized for use in HTS. The major disadvantage of this approach is the price of radioligands that are typically used in these assays and inconvenient work with radioisotopes. As for non‐radioactive alternatives, there are also fluorescence‐based ligand binding assays employing e. g., fluorescence polarization. Such methods have been successfully used for screening of 5‐HT3,^[5]^ insect ryanodine receptor^[6]^ or hERG channel^[7]^ modulators.

Functional methods could be further divided into direct and indirect. The only method that directly measures the current created by the flow of ions through the target channel is the electrophysiology technique patch clamp. This method is still considered as the gold standard for ion channel studies as it provides unbiased data. Manual patch clamp suffers from extremely low throughput, and it is used mainly for detailed, mechanistic studies of individual active compounds or for final hit validation. Also, it is useful in systems that do not have a high channel expression, like e. g., primary cells or tissues as this method is very sensitive. In the last few years, automated electrophysiology approaches have been developed, that increased throughput significantly. However, these systems are still not yet able to work with libraries of millions of compounds, they are expensive to operate and require highly qualified operators.

Alternative label‐free detection techniques that enable researchers to observe cellular responses without the need for exogenous labels or dye include digital holographic microscopy (DHM),^[8]^ multi‐electrode arrays (MEA),^[9]^ atomic absorption spectroscopy (ICR 12000 reader by Aurora)^[10]^ or impedance‐based platforms employing dielectric spectroscopy (DS)^[11]^ or real‐time cell analysis (xCELLigence®RTCA CardioECR).[^12^, ^13^] Similarly to patch clamp, these techniques require sophisticated, costly instrumentation, in some cases accompanied with expensive consumables. This all hinders their widespread use.

This review is focused on cell‐based indirect functional methods that employ fluorescent probes.

## 2 Fluorescence‐Based Methods

Fluorescence‐based methods involve the use of fluorescent probes to monitor changes in ion channel activity, providing a rapid and quantitative means to identify compounds that modulate ion channel functions. These assays can be automated and performed in 384‐ or 1536‐well plate formats, which makes them suitable for ultra‐high throughput screening of large compound libraries. They are also cost effective, when miniaturized, and they do not require special instruments and highly skilled operators. Importantly, they still provide quality data when properly validated. To exemplify this, we evaluated the effect of several reference compounds on intracellular calcium fluctuations in spontaneously beating neonatal rat cardiomyocytes using two independent assays, one of which was based on EarlyTox® fluorescent probe^[14]^ and the other was based on impedance measurement (RTCA). We concluded that the performance of both assays was equivalent (Table 1). Moreover, fluorescence‐based data were achieved with use of a common multimode plate reader (TECAN Spark®) and with significantly (approximately hundred times) lower costs (0.05 € per sample).

**Table 1:** Comparison of RTCA and fluorescence‐based analysis of Ca^2+^ intracellular fluctuations for monitoring contraction frequency of neonatal rat cardiomyocytes and their response to the treatment with cardiotoxic compounds. Impedance changes caused by cell movements were recorded with use of xCELLigence® RTCA CardioECR. The ion flux fluorescent probe EarlyTox® was employed to measure intracellular calcium ions.

Compound	Ca^2+^ (EarlyTox®)	Impedance (RTCA)
Verapamil	20 (10–80)	40(30–70)
Isoproterenol	0.8 (0.2–1)	6(2–20)
E‐4031	7(6–9)	30(20–40)
Amiodaron	1100 (1000–1300)	900(200–1300)
Doxorubicin	1700(1000–2100)	6300(5800–6600)
Propranolol	90(40–100)	200(20–300)

The table shows EC_50_ values (50 % of the maximal oscillation frequency of either calcium release or cell spontaneous contractions measured with RTCA, caused by selected compounds), expressed as the arithmetic mean, along with their confidence intervals at a significance level of 0.05 (all in units of nM). The measurements were conducted in triplicate during two independent experiments.

Moreover, some of the fluorescence‐based assays can be multiplexed, e. g., ion flux together with membrane potential measurements, providing more comprehensive data in a single assay.

In some cases, these methods offer real‐time information about ion channel activity, providing data about the kinetics and the dynamics of ion channel function. When optimized, most of the assays can be sensitive, however not as sensitive as manual patch clamp, which allows to read the currents from single channels.

Naturally, fluorescence‐based methods are not flawless, and their use brings some disadvantages that must be considered. For example, the probes can sometimes interact with ion channels by themselves, e. g., positively modulate them,^[15]^ which can lead to artefacts. The membrane potential probes are not ion specific, and concurrent activation of multiple ion channels may interfere with the measurement of the target channel. In most cases, fluorescent probes are toxic upon prolonged exposure, and the cells typically cannot be further cultivated after the experiment. Also, the temporal resolution provided by fluorescent probes may not be sufficient for the fast‐reacting ion channels, leading to incomplete or inaccurate measurement of their dynamics.[^16^, ^17^] Some fluorescent probes like sodium indicator SBFI^[18]^ or Fluo‐4,^[19]^ are sensitive to pH changes, which can confound ion channel measurements, especially in environments where pH fluctuations occur, such as in acidic organelles or during endocytosis. Furthermore, fluorescence methods struggle to resolve the opening, activation, or inactivation of the target ion channel, as well as any sub‐steps that may occur within these processes.

It is important to realize that fluorescence‐based techniques cannot fully substitute for the electrophysiology and thorough validation needs to be done to confirm the specificity of the observed changes of fluorescence signal (i. e., that the signal corresponds only to a desired ion channel activity). See also 3.12 and 3.13.

Fluorescence‐based functional methods for identification of ion channel modulators include (1) flux‐based assays using ion specific fluorescence probes and (2) assays using dyes sensitive to the change of membrane potential.

### 2.1 Membrane Potential Sensors

As ion channels allow for ion flow through membrane, the membrane potential changes. The ion channel activity can be indirectly measured in the cells using fluorescent dyes that detect the change in membrane potential. However, many confounding factors can contribute to the observed signal attributed to the membrane potential change, e. g., non‐specific binding of the dye, cellular uptake, or photobleaching. Therefore, proper controls must be included in the workflow.

Membrane potential fluorescent dyes can be divided into two major groups depending on their mechanism of action: (1) the dyes that change their fluorescence by potential‐depended accumulation and redistribution within the cell membrane, and (2) the dyes that undergo Förster resonance energy transfer (FRET). Besides these, there are also protein sensors of membrane potential available (see section 2.1.3.).

#### 2.1.1 Membrane Redistribution‐Based Dyes

**Figure 1:**
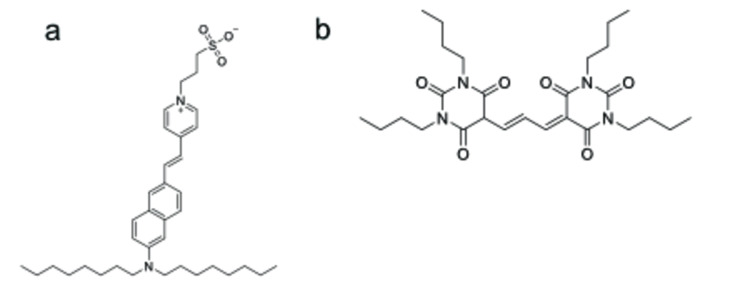
a) Structure of di‐8‐ANNEPS, an example of fast response membrane potential probe. b) Structure of DiBAC4(3), an example of slow response membrane potential probe.

Potentiometric probes that accumulate in the membrane typically include chemical moieties such as styryl dyes (di‐8‐ANNEPS, Figure 1), the cationic carbocyanines and rhodamines, oxonols or Merocyanine 540. They differ in their response mechanism and kinetics, toxicity, accumulation in cells or photostability. Their main disadvantage is their nonspecific binding to all membrane systems in the cell, which can lead to assay artefacts.

*Fast response probes*, often based on styryl dyes (Figure 1a), bind in the cell membrane and respond to a change in electric fields by changing their electronic structure and consequently altering their fluorescent properties. The process is fast enough to detect millisecond potential changes, which is suitable for tracking the neuronal or cardiac cells and tissues. The signal to noise ratio is often small–only 2–10 % fluorescence change per 100 mV. The sensitivity toward rapid changes in membrane potential can be improved using probes based on FRET, instead. See 2.1.2.

*Slow response probes* also bind to cell membrane, but they change their transmembrane distribution and fluorescent properties as the membrane potential changes. These dyes include mostly lipophilic, negatively charged oxonols (Figure 1b). Their fluorescence in aqueous solution is low but increases rapidly upon binding to the hydrophobic part of a membrane. The change in fluorescence is typically 1 % per mV. When the cell membrane is depolarized (for example when target ion channel is activated or inhibited), the probe moves into the cell and binds to intracellular membranes, which causes drop in fluorescence. These dyes are suitable for slow reacting channels or channels that are not easily desensitized in non‐excitable cell systems.[^20^, ^21^]

#### 2.1.2 FRET‐Based Membrane Potential Probes

The FRET‐based sensors are typically represented by the pairs of dyes – negatively charged membrane binding oxonols like [DiSBACn(3)] acting as FRET acceptors, and coumarin‐tagged phospholipids (CC2‐DMPE) as FRET donors that are bound to the outer leaflet of the membrane. The FRET signal changes depending on membrane hyperpolarization or depolarization. The advantage over non‐FRET sensors is, that the FRET donor molecules are fixed specifically in the cell membrane, therefore the signal comes only from that and not from other cell compartments. The FRET is rapid, and therefore particularly suitable for fast reacting channels. There is one limitation, though, that arises from the nature of the dyes. As the oxonol molecule is negatively charged, the dye itself may change the membrane potential when used at high concentration and thus contribute to the nonspecific signal, especially if the signal coming from a target is low. Therefore, dye concentration must be carefully optimized for a specific system.[^20^, ^21^, ^22^, ^23^]

#### 2.1.3 Genetically Encoded Protein Sensors of Membrane Potential

Voltage‐sensitive dyes are known for their simple use and reasonable sensitivity in assaying ion channels. However, their utility is hampered by issues such as non‐specific cell staining and limited permeability to some cell types e. g., primary cells. To address these limitations, researchers have turned to genetically encoded fluorescent sensors of membrane potential.

These sensors offer the advantage of cell type specificity, which can be achieved through various means. For instance, lentiviruses can selectively infect certain cell types^[24]^ leading to targeted expression of the fluorescent protein voltage sensors. Alternatively, various promoters can be used to drive expression in particular cell types.^[25]^ Also, protein sensors are better for long‐term experiments as these are usually not toxic.^[26]^

Despite the advancements in the field, challenges remain. Engineering and optimizing protein sensors can be a challenging and time‐consuming process, requiring specialized expertise and resources. Similarly to small molecule probes, depending on their design and mechanism of action, protein sensors may interfere with endogenous cellular processes. Also, protein sensors typically possess slower response time compared to small molecule dyes. In some cases, only small fluorescence changes are yielded during channel activation. The examples of protein voltage sensors include ArcLight (35 %), ASAP (49 %), QuasAr2 (90 %), VSFP2 (4–13 %), Mermaid (13–40 %), BeRST (32 %), SPARC (0.5 %), Archon1 (48 %), FlicR (2.5 %).[^27^, ^28^, ^29^, ^30^, ^31^, ^32^] The numbers in brackets are fluorescence changes per 100 mV in %.

### 2.2 Ion Flux Assays

Electrochemical gradient‐driven transport of ions is referred as ion flux. Assays that detect these or similar ions can be used to study ion channel activity.

Historically, radiolabeled ions were used to study a flux through specific channels. Later, fluorescent molecules that specifically bind physiologically relevant ions and change their excitation/emission spectra upon the binding have emerged. Chemically, they are either fully synthetic small molecules, or naturally occurring proteins that were genetically modified. The latter can be expressed directly in the cells of interest containing the target ion channel.

In these assays, researchers can directly detect the physiological ion of interest or alternatively, they may opt for surrogate ions.

#### 2.2.1 Small Molecule Probes for Ion Flux Assays

Fluorescent ion flux probes are small molecules that detect intracellular ions. They could be divided into two main groups: ratiometric and non‐ratiometric (Table 2). Ratiometric probes change their fluorescence emission spectrum at two different excitation wavelengths after ion binding. The changes in ion concentrations are reported as ratios of these two fluorescence values. These probes give internal calibration, which eliminates variations in probe concentration, optical path length (cell thickness), bleaching and illumination intensity. This allows for a more precise measurement of alterations in ion concentration, capturing even minor changes with greater accuracy. However, these probes often suffer from lower sensitivity.^[33]^

**Table 2:** Examples of commonly used fluorescent ion flux probes for detecting ions (including surrogate ions) within the cells and their fundamental properties. These probes are suitable for high‐throughput screening. The information was retrieved from the websites of the individual manufacturers.

Ion	Probe	Ratiometric	Notes	References
Calcium	Fluo‐8 AM	No	High fluorescence intensity, selectivity, rapid response kinetics, photobleaching, washing steps may be avoided	[35–37]
	Fura‐2 AM	Yes	UV excitation, dual wavelength measurement, sensitive to pH changes, washing steps necessary	[38,39]
	Fura‐8 AM	Yes	UV excitation, improved selectivity and fluorescence intensity over Fura‐2 AM	[40]
	X‐Rhod‐1 AM	No	Red‐shifted emission, suitable for deep tissue imaging, good signal to noise ratio, photostability, high affinity for calcium, washing steps necessary	[41]
Sodium	CoroNa™ Green AM	No	High sensitivity, sufficient specificity, good cell permeability, response to broad sodium concentrations	[42,43]
	SBFI AM	Yes	Low signal to noise ratio, low specificity, dual wavelength measurement, UV excitation, low fluorescence quantum yields	[44,45]
	Sodium green AM	No	Lower sensitivity then CoroNa™ Green AM, limited specificity, photobleaching, worse cell permeability	[44,45]
	CoroNa™ Red AM	No	Red shifted excitation, mitochondria targeted, good specificity	[46]
Potassium	IPG‐4 AM	No	High sensitivity, good specificity	[47]
	FluxOR™ dye (Surrogate ion Tl^+^)	No	High sensitivity and specificity	[48,49]
Chloride	PBFI AM	Yes	Low specificity, low fluorescence quantum yields	[50]
	MQAE	No	High sensitivity, high fluorescence quantum yield, washing steps necessary, low membrane permeability	[51,52]
	SPQ	No	Moderate sensitivity, limited specificity, low membrane permeability	[52–54]
	Dihydro MEQ	No	High membrane permeability, spontaneous oxidation	[52,55]

Non‐ratiometric ion probes change their fluorescence intensity at a single wavelength upon binding to target ion. The change in the intensity is proportional to the intracellular ion concentration. The sensitivity depends on a specific probe. For example, calcium probe Fluo‐8 is more sensitive than ratiometric probe Fura‐2.^[33]^ Non‐ratiometric sensors are less demanding for instrumentation, as only a single wavelength is measured. The choice between ratiometric and non‐ratiometric probes depends on the specific requirements of the experiment and the characteristics of the intracellular ion dynamics being studied. Both types of probes have their strengths, and the selection of the most suitable probe is based on factors such as specificity, sensitivity, fluorescence quantum yield or experimental design.

Sometimes, fluorescent probes sensitive to non‐physiological ions are used to facilitate the measurement of ion channel activity. This is often necessary because the ideal probe for the physiological equivalent may not be available for optimal detection. These so‐called surrogate ions are often employed in assays to mimic the behavior of the target ion while increasing sensitivity and lowering noise since they are not naturally present in the cells. For example, Rb^+^ or Tl^+^ are often used as surrogate ions for potassium ions (K^+^).^[34]^

For measurements in live cells, a cell‐permeable version of the probe is typically needed, if the probe does not pass the membrane by itself. For the details, see sections 3.5–3.7.

#### 2.2.2 Protein Sensors for Ion Flux Assays

Another significant category of molecules used for detecting ions comprises protein sensors. Typically, these sensors are directly encoded within the cells or cellular compartments that express the targeted ion channel, which is also their primary advantage. This can be accomplished with viral vectors or cell‐specific promoters. Another benefit of this approach is the cost‐effectiveness, as the probe is generated within the cell. However, challenges often arise regarding the sensitivity and reaction speed of these probes. With certain fluorescent proteins, photobleaching may occur after prolonged exposure to excitation light. To address this issue, strategies such as enhancing sensor expression, reducing excitation light exposure, optimizing emission filters to maximize fluorescence capture, and using sensitive detectors can be employed. Fluorescence microcopy recognizes another well‐established strategy to mitigate photobleaching and reducing background signal – i. e., using long‐wavelength two‐photon excitation of the dye. So far, this technique has not been implemented into HTS workflows due to the lack of adequate instrumentation and thus poses challenges for further technical development.

As for the fluorescent protein sensors, there is a commercially available Premo™ Halide Sensor (Molecular Probes™), which is a YFP (Venus)‐based probe combined with the BacMam baculoviral delivery system that detects chlorides. This is notably advantageous for HTS due to its simplicity, absence of washing steps, and efficiency. Stable cell lines as well as difficult‐to‐transfect cells (e. g., primary cells) can be generated using this system.^[56]^ Moreover, by adhering to the manufacturer‘s protocols transduced cells can be prepared frozen and “assay‐ready”. This represents an elegant way to uncouple cell maintenance and preparation from screening. According to the manufacturer, assay‐ready cells are guaranteed to perform as early as 4 hours after the thawing.^[56]^

Other examples include calcium sensors GcaMP and its modifications^[57]^ or Cameleon,^[58]^ potassium sensors GEPII^[59]^ or GINKO1,^[60]^ chloride indicators Clomeleon^[61]^ or ClopHensor.^[62]^ Protein sodium sensors have not yet been published.

The major differences of the membrane potential and ion flux approaches the are summarized in Table 3.

**Table 3:** Comparison of ion flux and membrane potential‐based assays for the use in HTS.[^20^, ^21^, ^22^, ^23^, ^63^]

Aspect	Membrane potential probes	Ion flux probes
Measurement type	Changes in membrane potential	Ion flux rates
Specificity	No specificity for ions, give complex information about membrane potential change	High specificity
Sensitivity	Depends on probe and cell type	Depends on probe and ion type
Applicability	Wide ‐ voltage‐gated channels, ligand‐gated channels, and ion exchangers	Similar to membrane potential probes, limited to existing probes specific for target ions
Interference	Nonspecific binding, sensitive to changes nonrelated to ion channels, like cell health	Less prone to interference
Dynamic response time	Fast reacting probes typically have quicker response time than ion flux probes, slow reacting probes typically react much slower	Some ion flux probes may exhibit slower kinetics due to the time required for ion movement and equilibration
Quantitative measurement	Challenging, needs proper calibration, difficult to obtain real membrane potential data	Quantitative measurement of ion flux rates when well calibrated
Real‐time monitoring	Yes	Yes
Dynamic range	Narrow	Wide
Signal to noise ratio	Lower	Higher

## 3 HTS Fluorescence‐Based Assay Development

High‐throughput screening (HTS) is considered the main method of compound identification in the drug development process. Ion channels are excellent targets for a wide range of health conditions. When optimizing cell‐based ion flux assays or membrane potential‐based assays for the use in HTS, there are numerous parameters to consider. This overview covers assay parameters that can be optimized to improve signal‐to‐background ratios, Z‐factors, minimum significant ratios, and other key statistical metrics. We also include data and anecdotal evidence demonstrating the effectiveness of manipulating these parameters to optimize the HTS assay in a variety of cell lines.

### 3.1 Cell Model

In the development and optimization of any cell‐based assay, everything starts and ends with the cells. Minor details such as growth media composition, confluency, passage number, cell detachment method or plating conditions can have enormous impact on the assay parameters. An important factor is the selection of a cell line with a high level of expression of the channel of interest to achieve significant and robust signals in fluorescence‐based assays.

Immortalised cell lines (usually HEK‐293 or CHO) stably overexpressing a specific ion channel are often used to initiate a screening campaign. However, high constitutive overexpression of certain ion channels may sometimes be harmful for the cells. The use of vector encoding a target ion channel under an inducible tetracycline or doxycycline‐responsive promoter (e. g. pcDNA4/TO plasmid^[64]^) can overcome this problem. The advantage of this approach over the use of channel inhibitors is the absence of washing steps that are always undesirable in HTS. Another trick that can be used to eliminate the toxic effects of ion channel overexpression is transient transfection. Although generally not as convenient as stably expressing cell lines, transiently transfected lines have also proven to be a viable alternative to stable cell lines also in the HTS settings. Several protocols are available for the preparation of transiently transfected cells. Popular lipid‐based methods can potentially compromise membrane integrity, which can affect the outcomes of membrane potential‐based assays. Other methods used for transient transfection ‐ often with increased efficiency ‐ include electroporation (Neon, MaxCyte, etc.) or baculovirus‐mediated expression.^[65]^ Several commercially available kits using baculoviral systems (e. g., BacMam system)^[66]^ are currently available. Cells produced by this technique can be frozen, and after thawing, used for the experiment without further manipulation.

In summary, the choice between transient and stable transfection in ion channel screening depends on the specific goals, experimental design, and constraints of the study. Both methods have their advantages and limitations (Table 4) that should be carefully considered when planning ion channel screening experiments. Finally, it is essential to include control cell line with low or no expression of target ion channel to evaluate potential interference.

**Table 4:** Practical aspects of transient and stable transfection of the ion channel of interest with specific focus on their use in HTS.^[67]^

Aspect	Transient Transfection	Stable Transfection
Efficiency	High, rapid expression of target ion channels, expression levels decline over time	Lower transfection efficiency, but stable expression over time
Flexibility	Short‐term experiments and rapid screening of ion channel modulators	Long‐term expression enabling prolonged screening studies
Variability	Expression levels can vary between experiments and different cell passages	More consistent and reproducible expression levels over time
Cell Toxicity	May induce cellular stress or toxicity, affecting assay robustness	Generally not toxic, may involve selection pressure and integration artifacts, potentially impacting cellular physiology
Throughput	Suitable for high‐throughput screening, may require more frequent optimization due to variability	Well‐adapted for high‐throughput screening, initial setup may be time‐consuming

In addition to transfected cell lines overexpressing the target ion channel, more physiologically relevant cell models, such as primary cells,[^68^, ^69^] iPSC‐derived cells,^[70]^ organoids^[71]^ or immortalized cell lines expressing the same channel endogenously are highly valuable. These can be used for studying deeper biological effects or for confirmatory studies. Interestingly, functional fluorescence‐based assays can also be performed in synthetic lipid bilayers, nanodiscs and liposomes, with the target ion channel reconstituted inside. These engineered artificial models have high potential in high‐throughput screening.^[16]^

Ion channels often consist of multiple subunits. Transfection of multiple subunits to form a functional ion channel can be a challenging task, as it involves coordinating the expression of multiple components to ensure proper assembly and function. We have summarized some general steps and considerations to help troubleshoot and optimize the transfection of multiple subunits to form an ion channel below.

One of the approaches to form a functional ion channel with the correct subunit composition in the cell is to co‐transfect plasmids encoding individual subunits. In this case, it is necessary to optimize their ratio. The use of different promoters with appropriate strength for each subunit is also an option. Another approach is to use co‐expression vectors that allow simultaneous expression of multiple subunits from a single plasmid, often containing internal ribosome entry sites (IRES) or 2 A sequences. To verify the correct expression and folding of each subunit during ion channel assembly, it is common to label each subunit with a fluorescent protein tag.[^72^, ^73^, ^74^, ^75^]

### 3.2 Cell Growth and Maintenance

Each cell line requires specific growth conditions that must be maintained when transitioning an assay to high‐throughput screening format. Nevertheless, receptor expression levels may vary depending on the cell passage number or due to the selection conditions used to maintain the cells. Engineered cell lines tend to lose expression of the target of interest over time, so using cells with low passage numbers may be critical. Some ion channels with large extracellular domains may be partially cleaved during incubation with trypsin or Accutase, so an alternative cell harvesting method (i. e., the use of a non‐proteolytic cell removal agent such as Versene) should be considered. A general practice is to monitor changes in functional activity over time.

### 3.3 Coating

**Figure 2:**
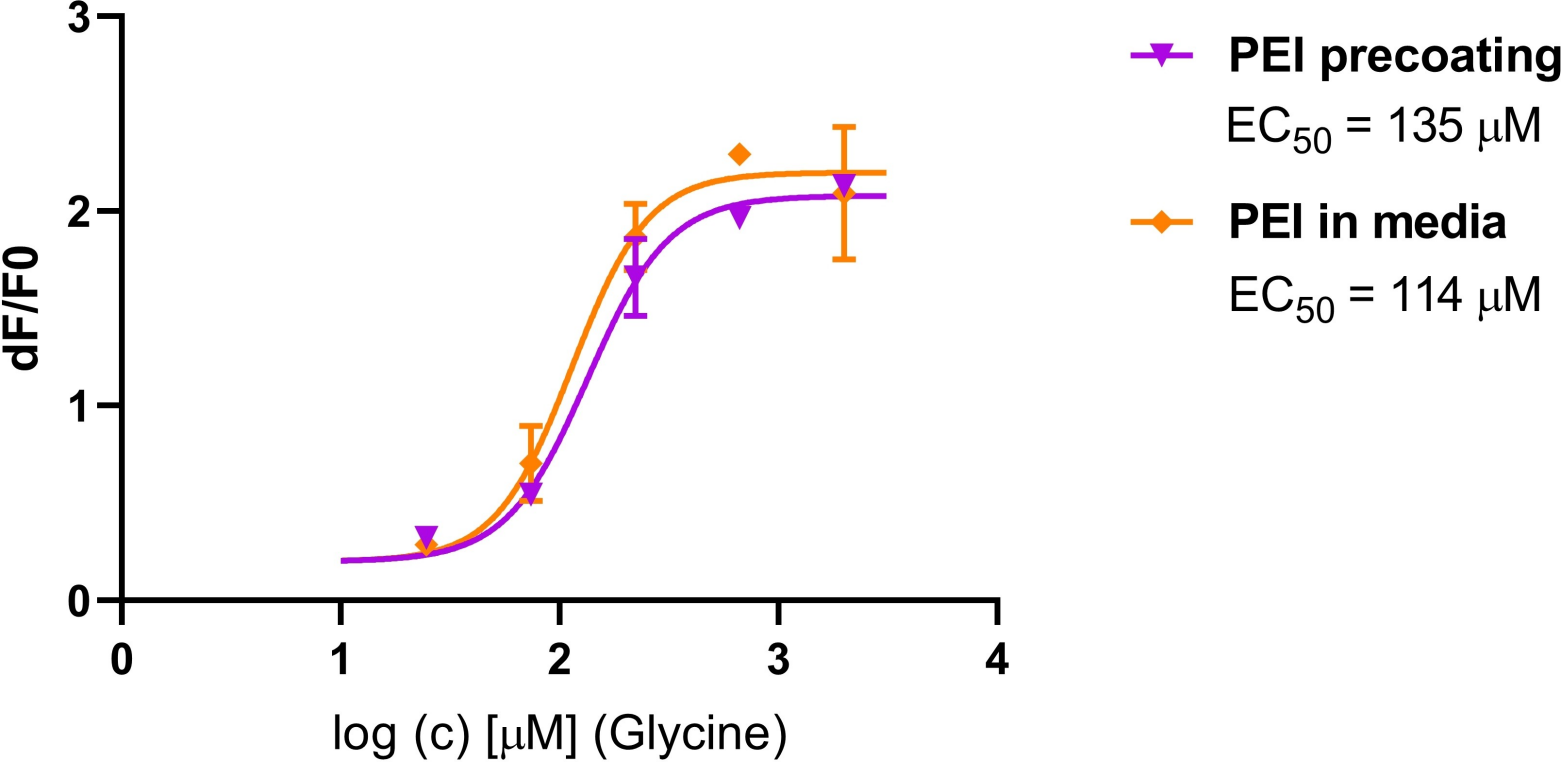
Comparison of glycine dose‐response curves measured on HEK 293 T cells expressing α3 subunit of glycine receptor (*Rattus norvegicus*) that were either seeded in a microwell plate precoated with PEI (20 μg/mL) or plated in culture media that already contained PEI (2 μg/mL). The data were measured using Membrane potential assay (FLIPR).

For some cell lines with weak attachment to culture vessels, such as HEK 293 T, chemical coating of multi‐well plates before plating the cells is used, especially if washing steps or other manipulations are included in the protocol. Some cells, e. g., primary neurons or iPSCs, require coating for proper growth/differentiation. Coating reagents include extracellular matrix proteins such as laminin, Matrigel, fibronectin or collagen, synthetic cationic polymers like poly – L – lysin, poly – D ‐ lysin, poly – L ‐ ornithine or gelatin. Typically, the plastic is coated prior to the experiment, however, we have found that the coating reagent can be added directly to the cell culture media during cell seeding without compromising the quality of the assay (Figure 2). This approach is particularly advantageous for HTS as it can save a lot of valuable time. It should be noted that the concentration of the coating reagent must be optimized in advance due to its possible toxic effects on the cells.

### 3.4 Cell Seeding

Each assay requires optimization of cell concentration (seeding density) and thus the amount of the target receptor. For instance, to obtain sufficient signal in membrane potential assays, it is often crucial to prepare a confluent cell monolayer. Modifying the conditions of cell plating can significantly impact the signal in a HTS, and these parameters are typically the first to be adjusted during HTS assay optimization. Both the plating media and cell density are strongly influenced by the cell type being utilized. Sometimes, reducing serum concentration in the media or decreasing the incubation temperature can enhance the signal, as temperature plays a role in channel trafficking to the plasma membrane.^[23]^

### 3.5 Dye Selection

The selection of a fluorescent probe or indicator is one of the essential steps in the assay development. For example, when using probes to measure ion flux, it is important to use a probe that is specific and has a high affinity for the ion of interest. This will minimize interference from other ions. The probe should be sensitive enough to detect even small changes in ion concentration and should have wide dynamic range and high signal to noise ratio. Another key factor for intracellular probes is their membrane permeability and stability. This strongly depends on cell line and culture conditions. Exclusion of the probe from the cell should also be minimized. Another factor is the photostability of the probe. It is preferable to use probes with low degree of photobleaching, especially if long kinetic measurements with long light exposure are performed. The fluorescence properties of the sensor molecule should also not interfere with other components in the system, such as the phenol red in the cell culture media, which is highly fluorescent when excited at 440 nm. The dye should not be cytotoxic for at least the time necessary to perform the measurement. For HTS, it is crucial to minimize the number of individual steps in the assay protocol. Therefore, if possible, it is better to use probes that do not require washing steps and the presence of the probe does not affect the measurement. Red‐shifted dyes are usually desirable because the long‐wavelength light (far‐red/near IR) used to excite them penetrates deeper into the tissue than short wavelength light (e,g, green) and is less phototoxic due to its lower energy.

The probe specifications should match the capabilities of the available detection equipment. For measuring fluorescent signals inside cells that are attached to the bottom of the plate, it is preferable to use plates with transparent (optical) bottom, and the signal should be acquired from the bottom of the plate to eliminate the interferences from the fluid above the cells. It is important to use UV transparent plates when using UV excitable dyes. For fluorescence assays, it is generally optimal to use plates with black walls.

### 3.6 Dye Concentration

Selecting an optimal probe concentration with sufficient affinity is essential to generate strong signals while avoiding saturation of the detector or dye. It is usually recommended to look for a probe/ion concentration combination that gives approximately 80 % of the maximum achievable signal for a specific probe/detector pair when the target channels are fully activated. Although an increase in dye concentration can widen the signal window to some extent, exceeding the upper detection limit of the instrument will only amplify background noise and ultimately reduce the signal window.^[23]^

### 3.7 Dye Loading

Probes detecting intracellular ions in living cells have to pass through the cell membrane and ideally remain inside the cell. There are several tricks to help these molecules in this direction. One of the most widely used is chemical modification introducing an acetoxymethyl group (AM), increasing the lipophilic nature of the probe and helping passage across the plasma membrane. Cytosolic esterases inside the cell hydrolyse the AM groups to form free acids that cannot escape from the cell, and the dye concentrates there. Conjugation of probes to AM often causes a decrease in the background signal due to a change or decrease in the measured fluorescence. The accumulation of AM esters in organelles such as the endoplasmic reticulum, vacuoles, or mitochondria can be problematic. However, this issue can be mitigated by using the dye at room temperature, for the shortest possible duration, and at the lowest effective concentration. Depending on the cell type, incubation time and temperature, the efficiency of AM groups hydrolysis will vary, leading to changes in the signal to noise ratio.

To further increase the signal, probenecid, sulfinpyrazone or Pluronic® F‐127 can be added to the assay buffer together with the AM‐modified probe.^[76]^ Probenecid serves as an inhibitor of organic anion transporters, which can enhance the efflux of anionic indicators and probes. This may result in a low signal and high background due to dye extrusion from cells. For cell lines expressing large amounts of organic anion transporters, such as CHO cells, the use of probenecid is recommended.^[77]^ Due to its toxicity, probenecid is added to the assay buffer only for the shortest time necessary and usually in concentrations of 1–2.5 mM.^[78]^ Pluronic® F‐127, a nonionic detergent, used to facilitate the dissolution of hydrophobic fluorescent probes (such as AM esters) in water‐based assay buffers improves the cell staining and thus the measurement signal.^[79]^

Depending on the application, it may be necessary to eliminate the fluorescent signal from outside the cell. This can be achieved either by washing the cells to remove probe residues or by using quenchers. In HTS, quenchers are preferred in order to minimize the number of manipulations and thus reduce well‐to‐well variability. Quenchers are molecules that can be used together with fluorescent probes. Unlike probes, quenchers do not enter the cell but remain in the media or assay buffer where they absorb fluorescence originating from residual dye and eliminate background fluorescence. Possible interactions of the quenchers with the receptors themselves should always be investigated and ruled out. Examples of quenchers used in fluorescence‐based assays to detect ion channel modulators are summarized in the patent EP2201367 A1 invented by Daniel Beacham and Kyle Gee. These include Tartrazine, Amaranth, Acid Red 37, Congo Red, Trypan Blue, Brilliant Black or Red 40.^[80]^

Generally, PBS and HBSS are used as assay buffers because their ionic composition can replace the culture medium and maintain the appropriate pH to support physiological processes in cells. Since most measurements are performed outside of standard cultivation conditions (without 5 % CO_2_), it is advisable to enrich the buffers with HEPES (5–50 mM), Tris (10–100 mM), or phosphate buffer (1–100 mM). Based on our laboratory experience, HBSS (with calcium and magnesium salts) supplemented with 20 mM HEPES allows for reproducible measurements of most fluorescence‐based assays for ion channel modulation outside the incubator for at least 3 hours in various cell lines, such as HEK 293 T and CHO.

### 3.8 DMSO Effect

It is particularly important to estimate the effect of the solvents used to dissolve assay components and tested compounds on the performance of the assay. The most commonly used solvent when working with living cells is DMSO. DMSO is universal solvent that dissolves both polar and non‐polar compounds, but can interfere strongly with membrane potential measurement.^[20]^

**Figure 3:**
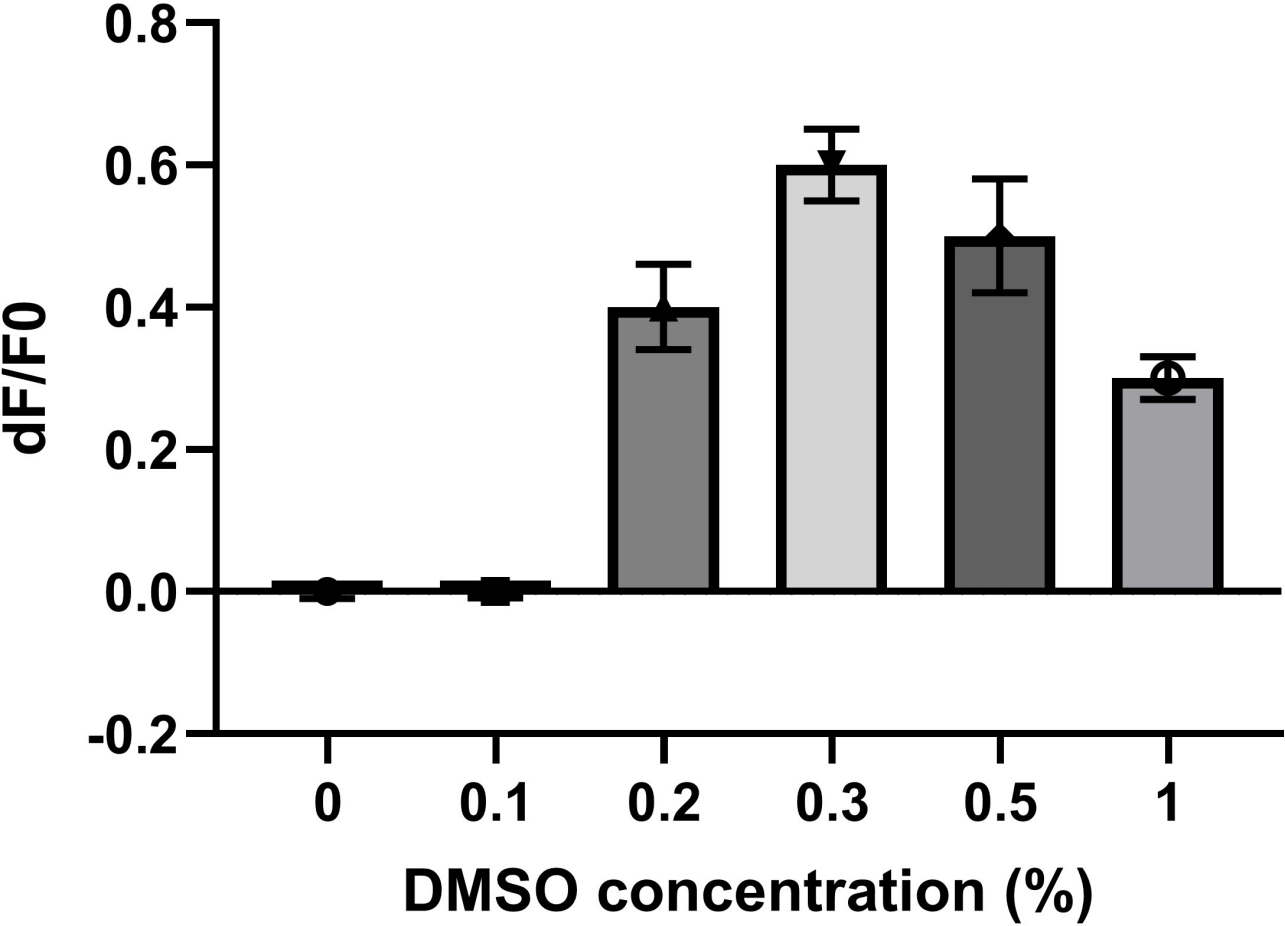
The effect of DMSO on a fluorescence‐based membrane potential assay (FLIPR). This assay was performed on CHO cells stably expressing α1β2γ2 GABAA receptors (B'SYS GmbH). The cells were loaded with Membrane potential dye (RED) and background fluorescence (F_0_) was measured. Then, DMSO at different concentrations was added and fluorescence was measured after 5 minutes (F). The signal is expressed as dF/F_0_.

Considering these facts, it is crucial to establish the highest applicable concentration of DMSO that must not be exceeded when implementing a new assay (Figure 3). Normalizing the DMSO concentrations across all wells in the assay and/or including DMSO controls is highly recommended.

### 3.9 Compounds Addition and Measurement

Currently, there are various instruments allowing rapid kinetic measurements of fluorescence or luminescence immediately after addition of the compounds, using high‐speed EMCCD cameras and pipette heads that can operate full 384‐/1536‐well plates simultaneously. Examples of such instruments include FDSS‐GX (Hamamatsu) and FLIPR (Molecular Devices). This is particularly beneficial for fast‐response ion channels. In the case of ion channels with slower responsiveness or for signals that endure longer, these instruments can be bypassed by employing a pipetting robot and a plate reader separately. Table 5 compares parameters of selected instruments designed for HTS of ion channel modulators.

**Table 5:** Some of the key parameters of available instruments used for fluorescence‐based HTS assays of ion channel modulators. The parameters were adopted from the manufacturer's websites.

Aspect	Tecan Spark	FlexStation	FLIPR Penta	FDSS‐GX
Liquid transfer	2 injectors	8/16 tip heads	96/384/1536 tip heads	96/384/1536 tip heads
Well plate format	96/384/1536	96/384	96/384/1536	96/384/1536
Camera	HR CCD	PMT	EMCCD/HS EMCCD	qCMOS
Readout	Absorbance/fluorescence/luminescence	Absorbance/fluorescence/luminescence	Fluorescence/luminescence	Fluorescence/luminescence

In our laboratory, we use the Echo 650 acoustic liquid handler (Beckman Coulter) to pre‐spot compounds to 384‐well plates and then utilize Certus Flex liquid dispenser (Fritz Gyger AG) to dissolve these compounds, either in assay buffer for agonist assays or, for example, in assay buffer with agonist when testing positive modulators.

Subsequently, prepared solutions of the test compounds are added to 384‐well culture plates containing pre‐seeded cells using the Bravo automated liquid handling platform (Agilent). This platform allows for adjustments to the speed and height of the solution addition based on the cell type. Depending on the assay, the volumes of individual components and test compounds added to the cells can be adjusted. As a standard procedure, we seed 20 μL of cell suspension and add 5 or 10 μL of the test compound solutions at the appropriate concentration.

The timing of addition of tested compounds (pretreatments) also requires optimization. In our experience, the simultaneous addition of the channel agonist and the tested allosteric modulator produces no discernible difference when compared to modulator pretreatment and subsequent agonist addition.

During the initial experiments, it is useful to measure the kinetics of the receptor's response to the reference modulators to assess the speed of the response and to gauge the stability of the signal after activation. Frequently, kinetic measurements can be replaced with endpoint measurements for the purposes of HTS.

### 3.10 Multiple Addition Experiments

Sometimes, it may be desirable and effective to measure potential activity of an unknown compound in several experimental modes (e. g., agonist, antagonist, positive modulator, or negative modulator) within a single experiment. Typically, the test compound is first added to the cells to observe potential agonistic or inhibitory effects. The measurement is followed by the second addition of a reference agonist EC_20_ to look for positive modulation, and finally the reference agonist at a final concentration of EC_80_ is added to evaluate potential negative modulatory/antagonistic effects.^[23]^

### 3.11 Calibration

In some cases, it is useful to determine precise intracellular ion concentrations in cells, such as when detecting calcium oscillations in neurons to study synaptic plasticity, which is involved in processes like learning and memory, or when studying the activity of cardiomyocytes through calcium influxes. Therefore, calibration with known ion concentrations inside living cells is performed before measurement. Ionophores, non‐ionic detergents, or patch pipettes are typically used to transfer ions across the membrane.

For example, calcium calibration buffers are typically composed of Ca^2+^‐EGTA and EGTA mixtures that generate given free calcium concentrations at specific pH, temperature, and ionic strength, although it is important to realize, that these values are just nominal. For the measurement of intracellular ion concentrations, cell membranes must be perforated or dissolved, so that the free calcium can bind to the intracellular probe. In the case of calcium, specific ionophore ionomycin, that binds calcium with high affinity and transports it into the cell, is typically used.^[81]^ Another, more universal reagent is digitonin.^[82]^ The calibration signal is then acquired with use of either fluorescence microscope or a plate reader.

With membrane potential‐based assays, it is possible to calibrate responses using extracellular K^+^ and parallel patch clamp measurements.^[83]^

### 3.12 Assay Validation

Each newly introduced assay must pass a validation test. It is crucial to validate an assay properly by being able to accurately discern significant levels of channel activity, as indicated by metrics such as Z‐factor,^[84]^ signal window,^[85]^ MSR^[86]^ or other HTS assay parameters. Equally important is the capability to measure potency and efficacy values for known classes of modulators targeting a specific ion channel. Ideally, optimized conditions for fluorescence‐based assays should be compared with electrophysiological measurements using a range of compounds known to affect the ion channel target. These compounds should exhibit diverse structures, potencies, and modes of efficacy.

### 3.13 Potential Artefacts in the Fluorescence‐Based Assays used in HTS

Like in any fluorescence‐based assay, the presence of compounds that interfere with the fluorescence measurements can lead to inaccurate positive or negative outcomes in the screening. The same applies for compounds exhibiting ionophore‐like properties, or detergents capable of affecting cell membrane integrity. Additionally, cell and assay conditions that induce receptor desensitization or internalization may also have impact on the results.

However, all of this is taken into account in HTS because the probability that some individual molecules from large compound libraries will interfere with the assay is high. Therefore, it is always necessary to perform further validation assays to confirm the preselected hits.

## 4 Summary and Outlook

With the increasing number of libraries of ion channel‐modulating compounds, the need for simple, inexpensive, reliable, fast and robust HTS assays is growing. Although the most direct and unbiased technique for estimating the channel activity is patch clamp, the automated systems are complex and expensive, requiring specialized equipment, skilled operators, and significant initial investment, which may be prohibitive for smaller research laboratories or screening facilities. Moreover, the throughput is lower compared to fluorescence‐based methods.

On the other hand, fluorescence‐based methods offer reasonable solutions for the first round of screening campaigns. They can be easily automated and adapted to 384‐ or 1536‐ well plate formats to be used in HTS. In addition, some of these assays can be multiplexed.

The disadvantage of fluorescence‐based methods compared to patch clamp is that they provide indirect measurements of ion channel activity, and the measured signal may not always correlate with channel function. For example, certain compounds or biological factors may interfere with fluorescence readouts, leading to false positives or false negatives.

In summary, fluorescence‐based methods are suitable for initial rounds of screening large libraries of molecules. Pre‐selected hits need to be further validated using less biased methods.

## Conflict of Interests

The authors declare no conflict of interest.

## Biographical Information

*Jan Voldřich received his Master's degree in Biochemistry from the University of Chemistry and Technology Prague the in 2019. He is currently pursuing his PhD studies in Biochemistry at the Institute of Organic Chemistry and Biochemistry of the Czech Academy of Sciences and the University of Chemistry and Technology Prague. His research focuses on biological activity of novel steroid compounds*.

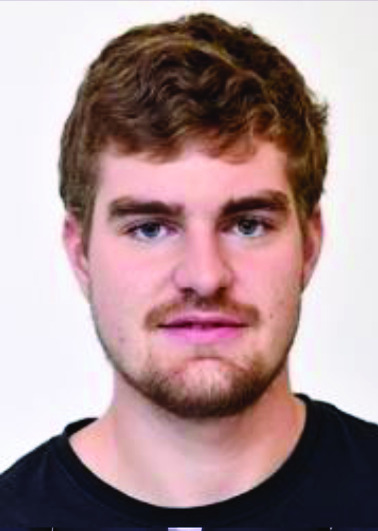

## Biographical Information

*Helena Mertlíková Kaiserová received her Master's in Pharmacy in 2003 and PhD in Biochemistry in 2007 at the Faculty of Pharmacy, Charles University in Prague. Since then she has been working at the Institute of Organic Chemistry and Biochemistry of the Czech Academy of Sciences where she currently leads the Biochemical Pharmacology Core Facility. She has been devoted to preclinical drug discovery ever since. She has been involved in the projects within the area of oncology, neuropharmacology and antimicrobials*.

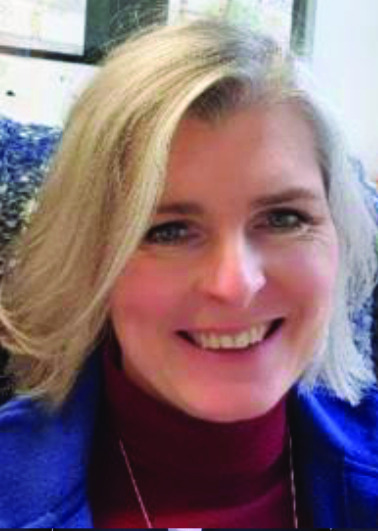

## Biographical Information

*Markéta Šmídková received her Master's degree in Biochemistry from the Charles University, Faculty of Science, Prague in 2001. In 2009, she finished her PhD study at Charles University, Faculty of Science, Prague. She currently works as a scientific assistant at the Institute of Organic Chemistry and Biochemistry of the Czech Academy of Sciences. Her research focuses on biological activity of novel steroid compounds*.

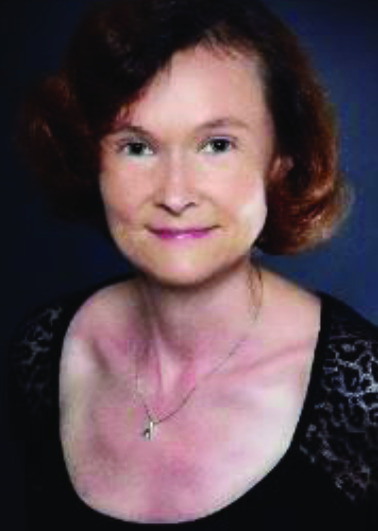

## Biographical Information

*Marika Matoušová received her Master's degree in Human Nutrition from the Slovak University of Agriculture in Nitra the in 2002. In 2008, she finished her PhD study in Biophysics at the University of South Bohemia in České Budějovice. She currently works as a scientific assistant at the Institute of Organic Chemistry and Biochemistry of the Czech Academy of Sciences, Prague. Her research focuses on in vitro assays development, HTS enzymatic screening of potential anti‐cancer compounds and the biological activity of new compounds on cell lines and primary cells*.